# Bioinvasion in a Brazilian Bay: Filling Gaps in the Knowledge of Southwestern Atlantic Biota

**DOI:** 10.1371/journal.pone.0013065

**Published:** 2010-09-29

**Authors:** Barbara L. Ignacio, Luciana M. Julio, Andrea O. R. Junqueira, Maria A. G. Ferreira-Silva

**Affiliations:** 1 Instituto de Bioquímica Médica, Universidade Federal do Rio de Janeiro, Rio de Janeiro, Brazil; 2 Instituto de Biologia, Universidade Federal do Rio de Janeiro, Rio de Janeiro, Brazil; Duke University, United States of America

## Abstract

**Background:**

Biological invasions are a major cause of global species change. Nevertheless, knowledge about the distribution and ecology of introduced species is regionally biased, and many gaps in knowledge exist for most developing countries.

**Methodology/Principal Findings:**

To study the zoobenthos on the hard substratum of the Ilha Grande Bay, a survey was conducted on both natural and artificial substrata at three depths and seven sites. The species recorded were classified as native, cryptogenic or introduced. Multivariate analyses were conducted to assess the prevalence of introduced species in these communities and to compare the distribution of species on natural and artificial substrata of this bay to identify possible discrepancies in habitat use. Of the 61 species, 25 were cryptogenic, 10 were introduced and 26 were native. Similar numbers of introduced species were found on both natural and artificial substrata, though the community composition was significantly different between them. We also compared the species composition of the Ilha Grande Bay survey to other inventories taken around the world. The highest similarities were found between the Ilha Grande Bay inventory and the Atlantic coastal region (Tampa Bay, USA and the Gulf of Mexico), American Samoa and Pearl Harbor (USA) inventories.

**Conclusions/Significance:**

This study presents the first published comprehensive list of hard substratum sessile marine invertebrate species in a Brazilian bay. The high percentage of cryptogenic species reveals gaps in both zoological records and information on introduced species for the Brazilian coast. The introduced species successfully colonized different sites in the Ilha Grande Bay, including both natural and artificial substrata. In addition, we find that artificial structures may not be good surrogates for natural rocky shores and may represent an ecological threat. Comparisons with other inventories suggest a history of broad-scale invasion, though more evidence is needed to support this conclusion.

## Introduction

Biological invasions, herein defined as the establishment of species beyond their historical range, have resulted from both human-mediated and natural pathways. Natural and prehistoric invasions most likely involve short-distance dispersal, whereas human vectors commonly involve saltatory transport of organisms and long-distance dispersal events [1]. Therefore, biological invasions alter the environmental connectivity in ways that is no longer driven only by biogeographic barriers and species behavior [2], [3]. In recent decades, shipping activities have been identified as the main source of species introductions in coastal estuarine and marine habitats. Ballast water, ballast water sediments and hull fouling are the main vectors associated with this pathway [2], creating unprecedented levels of biological exchange.

Anthropogenic influences extend to coastal landscapes. The increased urbanization of coastal areas has resulted in the loss of natural habitats, in particular reducing the availability of rocky shores. However, artificial structures such as moorings, piers, breakwaters and seawalls are becoming increasingly common in coastal areas and represent important sources of available substratum [4]. The characteristics of the substratum affect the colonization patterns, and these patterns impact the subsequent benthic hard substratum communities in different ways, as reported by several authors, eg. [5]–[13]. Although the intrinsic characteristics of the substratum are of great importance, and acknowledging the often critical role of predation in structuring communities, space is often the main limiting factor for epibiota [14]–[17]. The provision of substratum by artificial structures helps mitigate this limitation. Despite this apparent positive effect for benthic communities, these structures do not necessarily act as good surrogates for natural rocky reef communities [18]–[21], and these anthropogenic structures may provide an opportunity for newly arrived species to become established. This is a particular concern for species that do not exhibit strong selectivity for specific artificial substrata, such as reported for the introduced corals *Tubastrea coccinea* and *T. tagusensis* [22]. The absence of evolutionary history of any species with the artificial substratum may negate many of the native species' potential advantages in the settlement process [12], [13], a factor that may increase the invasibility of coastal areas.

Museum data collection, survey efforts and descriptive ecological studies are essential steps in the research of community ecology and are often the only predictive tools available for bioinvasion management, particularly in poorly studied estuarine and marine coastal areas. Analyses of the geographic range of different species are also valuable. When there is no plausible evidence supporting the classification of a species as native (i.e., detected inside its original range of geographic distribution) or introduced (i.e., detected beyond its original range of geographic distribution), the species should be considered cryptogenic - until further revision [23].

Our knowledge of the distribution and ecology of invasive species in the world contains a regional bias. This bias is exemplified by the 28 unsolicited papers on invasion ecology analyzed by Richardson & Pyšek [24] that comprise information from Europe (12 papers), Australasia (6), North America (4), Africa (3), Asia (1), the Pacific Islands (1) in addition to one global overview.

Some biodiversity and biological invasion studies of South American locations have been published. For the southwestern Atlantic coast, four inventories of introduced marine species from Argentina and Uruguay [25] and Brazil [26]–[28] have been published. However, to our knowledge, a comprehensive regional survey of sessile marine hard substratum invertebrates on the Brazilian coast has not been published.

The aims of this study were as follows: (i) to present a comprehensive list of the sessile hard substratum marine invertebrate species in a Brazilian bay, (ii) to analyze their biogeographic distribution to classify species as native, cryptogenic or introduced, (iii) to assess the prevalence of introduced species among sites with different types and/or levels of exposure to human disturbances (harbor, marinas and islands), (iv) to compare the distribution of species on natural and artificial substrata of this bay to identify possible discrepancies in habitat use and (v) to compare the species similarities between the Ilha Grande Bay survey and other inventories taken around the world, considering both geographic distance and environmental conditions (i.e., temperate or tropical areas).

## Methods

The Ilha Grande Bay in southeastern Brazil (22°55′ to 23°15′ S and 44°00′ to 44°43′ W) covers an area of about 1,000 km^2^ and contains roughly 350 islands surrounded by shallow water (typically no more than 8 m in depth). It is an oligotrophic ecosystem with a small number of local and restricted domestic sewage discharges near major urban areas, a nuclear power plant, some marinas, a port area and one of the largest oil terminals in Brazil. Man-made structures are common on the coastline of this bay. Despite the human activities, the Ilha Grande Bay is not considered a heavily impacted ecosystem [29], and it supports a number of critical fisheries and marine resources.

**Figure 1:**
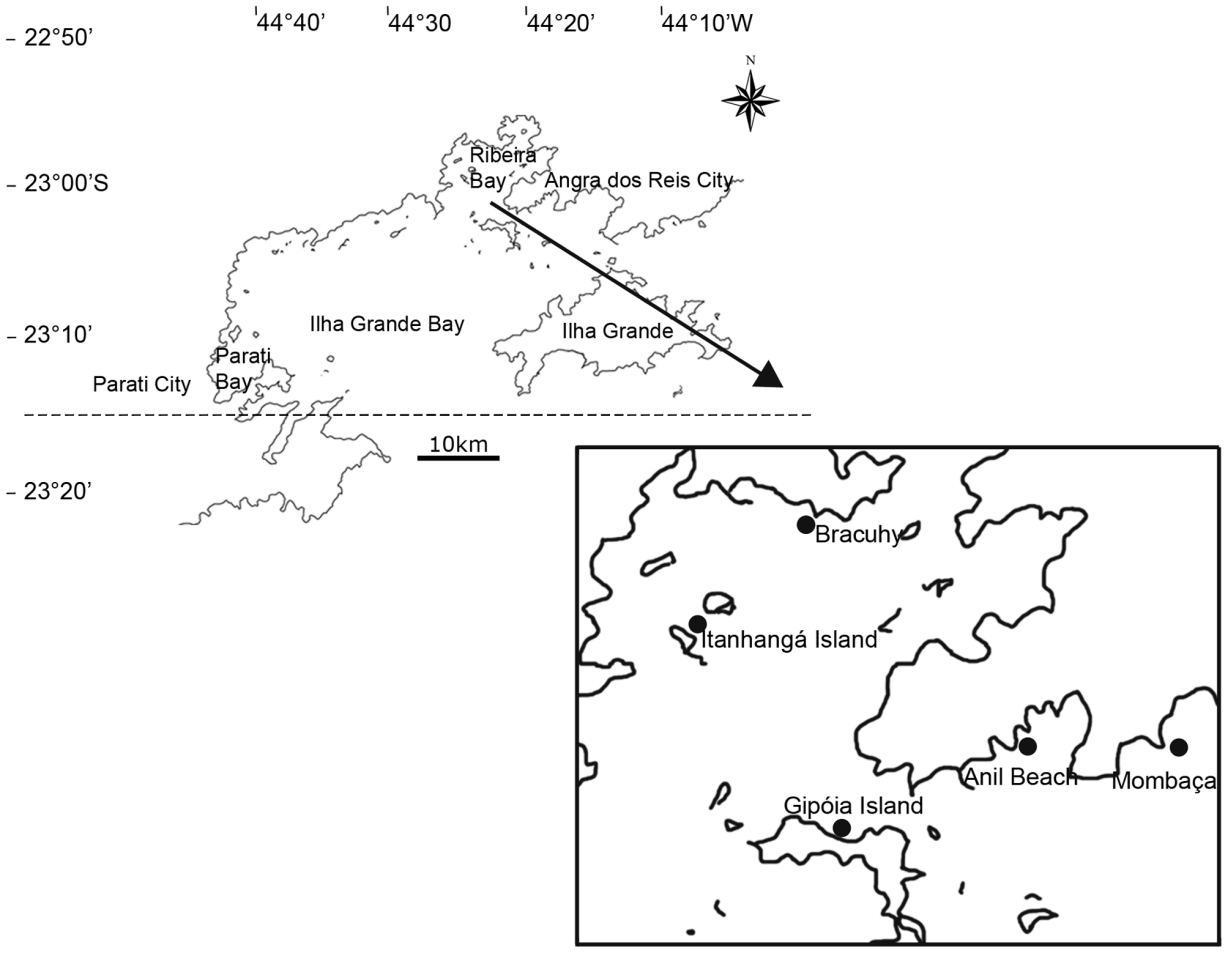
Map of the Ilha Grande Bay and the analyzed sites: Anil Beach, Mombaça, Bracuhy, Gipoia Island and Itanhangá Island.

Sites with different types and/or levels of exposure to human disturbances were chosen for analysis. Each site contained both natural and artificial substrata separated by less than 50 m. The natural substrata sampled were rocky shores with similar slopes. To avoid collecting samples affected by different resuspension and sedimentation conditions, data was always collected at least 1.5 m from the bottom surface. The sites analyzed were Anil Beach, Mombaça, Bracuhy, Gipoia Island and Itanhangá Island. Anil Beach, where the Port of Angra dos Reis is located, is surrounded by a major urban area (Angra dos Reis city) and is a site of domestic sewage discharge [30]. Mombaça is a new urban area in this bay that is marked by a large number of summerhouses and private piers, most of which were built on rocky shores. Bracuhy is site of the largest marina in South America (Marina Bracuhy). There are no records of organic pollution [30] at Gipoia Island or Itanhangá Island (Figure 1).

In September 2004, a survey of both natural and artificial substrata was conducted at depths of 0.5, 2.0 and 5.0 m from the mean low water (MLW) level at the sites described above (Table 1). At each site/depth/substratum, four samples were obtained through SCUBA diving. For each sample, 0.10 m^2^ quadrats were placed randomly on the substratum, and all organisms were carefully scraped off into a 0.5-mm nylon mesh bag. The replicates of each site/depth/substratum were kept as separate samples and preserved in 4% buffered formaldehyde for further sorting and taxonomic identification. Porifera, Cnidaria (Anthozoa), Mollusca (Bivalvia and sessile Gastropoda-Vermetidae), Bryozoa, Arthropoda (Maxillopoda) and Chordata (Ascidiacea) were the target groups. The species were classified as native, cryptogenic or introduced according to their origin based on a literature review, previously developed criteria [23], [31] and assistance from taxonomists.

**Table 1:** Summary of the sampling design.

	0.5 m	2.0 m	5.0 m
Anil Beach	n - a	a	a
Mombaça	a		
Bracuhy	a		
Gipoia Island	n - a	n	n
Itanhangá Island 1	n	n	n
Itanhangá Island 2	n	n	n
Itanhangá Island 3	n - a		

(**n**): natural substratum; (**a**): artificial substratum; (**0.5 m**): 0.5 meter deep; (**2.0 m**): 2.0 meters deep; (**5.0 m**): 5.0 meters deep. Total number of samples: 72.

Multivariate analyses were conducted to: (i) assess the prevalence of introduced species on the sessile marine invertebrate communities analyzed and (ii) compare the distribution of species on natural and artificial substrata of this bay. As the sampling procedure did not exclude possible interactions of depth and site with the type of substratum, evaluations of these factors were also included in the analyses. All multivariate analyses were performed using PRIMER (Plymouth Routines In Multivariate Ecological Research) v5® software. The community at each site, depth and type of substratum was represented by species frequencies. The communities were graphically presented in two-dimensional ordination plots by non-metric multidimensional scaling (nMDS) using the Bray-Curtis measure of similarity. One-way ANOSIM with pair-wise comparisons (which is an approximate analogue of standard analysis of variance based on similarity matrices) was conducted to formally test the factors. The Similarity Percentage Procedure (SIMPER) was used to identify the species that contributed most to the similarities within groups and dissimilarities between groups.

The species similarities between the Ilha Grande Bay survey and other inventories were assessed using the Sørensen similarity index [32], taking into account both geographic distances and environmental conditions. Geographic distances from the Brazilian site to each of the other sites were estimated using Google Earth^©^ software. Minimum distances were considered for all sites except for American Samoa, Chile, Port Philip Bay (Australia) and Tasmania. For these sites, we also considered the most probable routes. The inventories used were obtained from seventeen peer-reviewed scientific publications and government reports representing several sites around the world. The selection criterion for these inventories was the availability of a data set for sessile zoobenthic hard substratum species.

## Results

This study presents the first published comprehensive survey of sessile invertebrate communities on hard substrata in the Brazilian coast. A total of 85 taxa were recorded, including 20 Porifera, 3 Cnidaria, 20 Mollusca, 24 Bryozoa, 6 Arthropoda-Maxillopoda and 12 Chordata-Ascidiacea. Porifera and Bryozoa were the phyla with lower taxonomic resolution. Altogether, 56 and 68 taxa were recorded in natural and artificial substrata, respectively. Table S1 shows the detailed distribution of all taxa recorded at the Ilha Grande Bay. Of the 85 recorded taxa, 61 were identified to the species level. Of these, 25 (41%) were considered to be cryptogenic, 10 (16%) were introduced and the remainder were native species. The species and their worldwide geographic distributions are presented in Table S2. The introduced barnacle *Balanus trigonus* was the only species recorded at all sites, depths and types of substrata. Of the species detected in two or more samples (site/depth), four species were exclusively recorded in natural substrata whereas 18 taxa were exclusively recorded in artificial substrata.

Introduced and cryptogenic species were found in all regions of the bay. Eight of the ten introduced species were recorded in both natural and artificial substrata, including the octocoral *Carijoa riisei*, the incrusting bryozoan *Schizoporella errata*, the bivalves *Isognomon bicolor*, *Perna perna* and *Lithophaga* (*Myoforceps*) *aristata*, the barnacles *Amphibalanus reticulatus* and *B. trigonus* and the solitary ascidian *Styela plicata*. The barnacle *Megabalanus coccopoma* was only recorded on artificial substrata. However, there are previous reports of this species on natural substrata [33]. The arborescent bryozoan *Scrupocellaria diadema* was recorded only on natural substrata.

**Figure 2:**
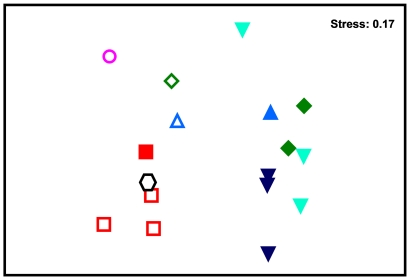
Two-dimensional nMDS ordination for the sessile invertebrate communities in Ilha Grande Bay. Empty symbols: artificial substrata; round symbols: natural substrata. Red square: Anil Beach, pink circle: Bracuhy, black hexagon: Mombaça, green diamond: Gipoia Island, dark blue upside-down triangle: Itanhangá Island 1, blue upside-down triangle: Itanhangá Island 2, blue triangle: Itanhangá Island 3.

The samples taken from 5.0 m at Gipoia Island only contained the anthozoan *Palythoa caribaeorum* and were not considered in the multivariate analysis. The similarity of species was not significantly different among depths (Global R = 0.191; *p*>0.05). However, nMDS analysis revealed a grouping of samples by sites that was confirmed by the ANOSIM test (Global R = 0.576, *p*<0.05). Pair-wise comparisons revealed significant differences in community composition between Anil Beach and the Gipoia and Itanhangá islands (A,G: R = 0.778, *p*<0.05; A,I1: R = 1.000, *p*<0.05 and A,I2: R = 0.889, *p*<0.05). Despite the site differences, the samples were grouped by the type of substratum as confirmed by the ANOSIM test (Global R = 0.462, *p*<0.05) (Figure 2). The species that contributed most to the similarity of the samples within the different sites and types of substratum sampled are listed in Tables 2 and 3 respectively. SIMPER analysis revealed an average dissimilarity of 69.11% between natural and artificial substrata. The introduced species *I. bicolor* (bivalve), *S. errata* (bryozoan), *A. reticulatus* and *B. trigonus* (barnacles) and the native bivalve *Pinctada imbricata* were the major contributors to this dissimilarity. The species *S. errata*, *A. reticulatus* and *B. trigonus* were more frequently found on artificial substrata.

**Table 2:** Summary of the three species that most contributed to the similarity within sites.

	G	I1	I2	I3	M	B	A
*Amphibalanus reticulatus* **				2^nd^			
*Balanus trigonus* **	1^st^	1^st^	1^st^	1^st^	1^st^	1^st^	1^st^
*Chama (Pseudochama) radians*	3^rd^						
*Choristodon robustus*		3^rd^					
*Crassostrea rhizophorae*					3^rd^		2^nd^
*Herdmania pallida* *	2^nd^						
*Isognomon bicolor* **		2^nd^		3^rd^			
*Mycale angulosa*						3^rd^	
*Petaloconchus varians*			2^nd^				
*Pinctada imbricata*			3^rd^				
*Schizoporella errata* **						2^nd^	3^rd^
*Scrupocelaria* aff. *reptans*					2^nd^		

Gipoia Island (**G**), Itanhangá Island 1 (**I1**), Itanhangá Island 2 (**I2**), Itanhangá Island 3 (**I3**), Mombaça (**M**), Bracuhy (**B**) and Anil Beach (**A**).

*cryptogenic species;

**introduced species. SIMPER analyses were performed for **G**, **I1**, **I2**, **I3**, **A**.

**Table 3:** Summary of the SIMPER results showing the three species that most contributed to the similarity within substratum types.

	natural	artificial
	ASm: 40.18	ASm: 39.55
*Balanus trigonus* **	1^st^	1^st^
*Crassostrea rhizophorae*		3^rd^
*Isognomon bicolor* **	2^nd^	
*Petaloconchus varians*	3^rd^	
*Schizoporella errata* **		2^nd^

**ASm**: average similarity of the group.

**introduced species.

**Figure 3:**
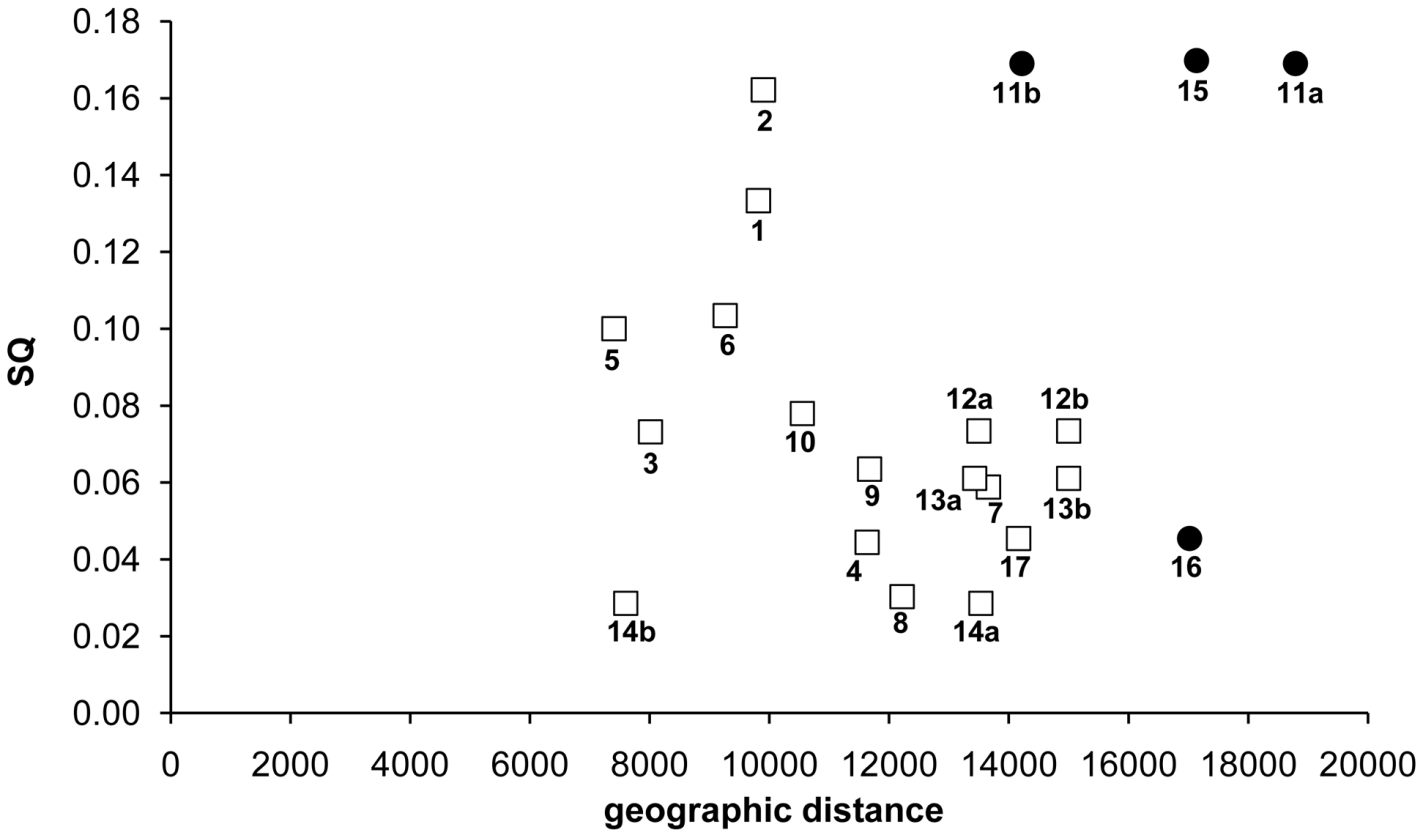
Geographic distance versus Sørensen similarity index for the analyzed inventories. Black circle: regions located in tropical regions; white square: regions located in temperate regions. (**1**) Gulf of Mexico [34]; (**2**) USA - Tampa Bay [35]; (**3**) European Coast - Atlantic Ocean [36]; (**4**) Israel Coast - Mediterranean Sea [37]; (**5**) Portugal - Azores [38]; (**6**) Mediterranean Sea [36]; (**7**) Caspian Sea [39]; (**8**) Baltic Sea [36]; (**9**) Black Sea [36]; (**10**) North Sea [36]; (**11**) American Samoa - Pago Pago Harbor, Fagatele Bay and National Park Coast [40]; (**12**) Australia, Victoria - Port Phillip Bay [41]; (**13**) Tasmania - Port of Launceston [42]; (**14**) Chile [43]; (**15**) USA, Hawaii - Pearl Harbor [44]; (**16**) USA, Hawaii - Lāna’i [45]; (**17**) USA, California - Elkhorn Slough [46].

Comparisons of the Ilha Grande Bay species records with other inventories are presented in Tables 4 and 5. The inventories from the Baltic Sea, Chile, the Israel coast, Lāna’i (Hawaii, USA) and California (USA) showed the lowest species similarities with the Ilha Grande Bay survey, whereas higher similarities were found with Pearl Harbor (Hawaii, USA), American Samoa, Tampa Bay (Florida, USA) and the Gulf of Mexico. The relationship between geographic distance and species similarity is graphically represented in Figure 3.

**Table 4:** Sampling methodology, total number of species available (**T**), numbers of cryptogenic (**C**) and introduced (**I**) species, number of species in common with the Ilha Grande Bay survey (**S**) and Sørensen similarity index (**SQ**) for the inventories analyzed in the present study.

Site\Reference	Methodology	T	C	I	S	SQ
Brazil, RJ - Ilha Grande Bay [present study]	DS	61	25	10	-	-
Gulf of Mexico [34]	LR	16	3	12	5	0.13
USA - Tampa Bay [35]	DS, LR	15	5	10	6	0.16
European Coast - Atlantic Ocean [36]	LR	23	NI	23	3	0.07
Israel Coast - Mediterranean Sea [37]	LR	31	NI	31	2	0.04
Portugal - Azores [38]	LR	21	8	13	4	0.10
Mediterranean Sea [36]	LR	57	NI	57	6	0.10
Caspian Sea [39]	LR	9	NI	9	2	0.06
Baltic Sea [36]	LR	7	NI	7	1	0.03
Black Sea [36]	LR	4	NI	4	2	0.06
North Sea [36]	LR	18	NI	18	3	0.08
American Samoa - Pago Pago Harbor, Fagatele Bay and National Park Coast [40]	DS, LR	12	1	11	6	0.17
Australia, Victoria - Port Phillip Bay [41]	NDS, DS, LR	50	8	42	4	0.07
Tasmania - Port of Launceston [42]	NDS, DS, LR	72	4	4	4	0.06
Chile [43]	LR	11	NI	11	1	0.03
USA, Hawaii - Pearl Harbor [44]	DS, LR	47	11	36	9	0.17
USA, Hawaii - Lāna’i [45]	NDS, DS	29	1	2	2	0.05
USA, California - Elkhorn Slough [46]	DS, LR	29	2	27	2	0.05

**NDS**: nondestructive sampling; **DS**: destructive sampling; **LR**: literature review; **NI**: non-informed data. The data are comprised of the following taxa: Porifera, Cnidaria (Anthozoa), Mollusca (Bivalvia and sessile Gastropoda), Bryozoa, Arthropoda (Maxillopoda) and Chordata (Ascidiacea).

**Table 5:** Comparisons between species recorded in the Ilha Grande Bay survey and their records in the others inventories analyzed in the present study.

	Taxon	1	2	3	4	5	6	7	8	9	10	11	12	13	14	15	16	17	18
**Porifera - Desmospongiae**	*Lissodendoryx isodictyalis*	**C**												**C**					
**Cnidaria - Anthozoa**	*Carijoa riisei*	**I**															**I**	**I**	
**Bryozoa - Gymnolaemata**	*Aetea anguina*	**C**												**I**					
	*Aetea truncata*	**C**															**I**		
	*Bugula neritina*	**C**										**I**	**I**	**I**	**C**	**I**	**I**		**I**
	*Savignyella lafontii*	**C**											**I**				**I**		
	*Schizoporella errata*	**I**											**I**				**I**		
**Mollusca - Bivalvia**	*Crassostrea rhizophorae*	**N**			**I**														
	*Hiatella arctica*	**C**					**C**												
	*Perna perna*	**I**	**I**	**I**															
**Arthropoda - Maxillopoda**	*Amphibalanus eburneus*	**C**			**I**		**I**	**I**	**I**		**I**	**I**					**I**		
	*Amphibalanus improvisus*	**C**			**I**			**I**	**I**	**I**	**I**	**I**							**I**
	*Amphibalanus reticulatus*	**I**	**I**	**I**		**I**		**I**					**I**				**I**		
	*Balanus trigonus*	**I**	**I**	**I**			**I**	**I**							**N**				
**Chordata - Ascidiacea**	*Botrylloides nigrum*	**C**	**C**	**C**															
	*Clavelina oblonga*	**C**					**I**												
	*Diplosoma listerianum*	**C**													**N**				
	*Microcosmus exasperatus*	**C**						**I**									**I**		
	*Phallusia nigra*	**C**				**I**		**I**					**I**				**I**	**I**	
	*Styela canopus*	**C**		**I**									**I**		**N**				
	*Styela plicata*	**I**	**I**	**I**										**I**					

**C**: cryptogenic species; **I**: introduced species; **N**: native species. **1**: Brazil, RJ - Ilha Grande Bay; **2**: Gulf of Mexico [34]; **3**: USA - Tampa Bay [35]; **4**: European Coast - Atlantic Ocean [36]; **5**: Israel Coast - Mediterranean Sea [37]; **6**: Portugal - Azores [38]; **7**: Mediterranean Sea [36]; **8**: Caspian Sea [39]; **9**: Baltic Sea [36]; **10**: Black Sea [36]; **11**: North Sea [36]; **12**: American Samoa - Pago Pago Harbor, Fagatele Bay and National Park Coast [40]; **13**: Australia, Victoria - Port Phillip Bay [41]; **14**: Tasmania - Port of Launceston [42]; **15**: Chile [43]; **16**: USA, Hawaii - Pearl Harbor [44]; **17**: USA, Hawaii - Lāna’i [45]; **18**: USA, California - Elkhorn Slough [46].

## Discussion

The wide distribution of the introduced species in the Ilha Grande Bay, in addition to the presence of several cryptogenic species, clearly demonstrates the high susceptibility of the system to biological invasions.

Several cryptogenic species were recorded in the Ilha Grande Bay. This high percentage of cryptogenic species reveals the lack of comprehensive historical zoological records for this region. Furthermore, information about the geographic distribution of several recorded species, particularly bryozoans and ascidians, is limited. Gaps in knowledge are common not only for the Ilha Grande Bay and other Brazilian regions but also for most other developing countries [47], [48]. This finding contrasts with records available for the coastal areas of the U.S.A. [49], [50], Australia [41], [51] and Europe [36], [52], [53]. In comparison, the percentage of cryptogenic species found in the Ilha Grande Bay is greater than that reported by Carlton in the Chesapeake Bay (Virginia, USA) at a time when there were no available systematic invasion studies [23]. This high number of cryptogenic species is of concern because it may lead to an underestimation of the number of introduced species and their impacts on natural and previously invaded communities [23].

Several species (34%) recorded in the Ilha Grande Bay survey were also recorded at least once in the seventeen inventories analyzed in the present study. This finding suggests that a considerable number of species have wide geographical distributions. Cosmopolitan distributions should be considered with caution as they may hide human-mediated historical introductions [23], [54], [55]. Wide-range geographic distributions may also hide erroneous taxonomic identifications [55], [56]. In the case of very common shipping-associated species, human-mediated introduction may be the more plausible cause of the wide geographical distributions recorded. This seems to be particularly true for some fouling species that were extensively recorded in the analyzed inventories, including the bryozoan *Bugula neritina*, barnacles of the genera *Amphibalanus* and *Balanus* and the ascidians *S. plicata* and *Phallusia nigra*. *Amphibalanus improvisus* was the most common species in the analyzed inventories. A well-documented example of shipping-related biological invasion was reported for this barnacle that was introduced via shipping into western North America in the mid-19th century. Mariculture of oysters represented an additional vector for this species [49].

Despite the presence of numerous species with wide geographical distributions, the biological composition similarities between the Ilha Grande Bay and the other analyzed inventories were low. Higher similarities were found between this bay and the tropical regions of Pearl Harbor (Hawaii, USA) and American Samoa, as well as with Atlantic coastal region inventories (Tampa Bay, Florida, USA and the Gulf of Mexico). The lowest inventory similarities identified were between the Ilha Grande Bay and the temperate regions (the Baltic Sea and Chile). This result was expected due to geographic distances and/or different climatic and environmental conditions. However, some findings are noteworthy, such as the higher levels of similarity found with the Pearl Harbor and American Samoa inventories and the lower similarity with the Lāna’i inventory.

Although these three regions are located in tropical zones, they are not geographically close to the Ilha Grande Bay. In fact, the Hawaiian Islands, where Pearl Harbor and Lāna’i are located, are the most isolated land areas in the world. However, the two regions with highly similar species compositions are historically important shipping routes. American Samoa has been involved in shipping for over one hundred years, and Pago Pago Harbor is a major harbor in the central South Pacific [40]. Similarly, Hawaii is considered to be the “crossroads of the Pacific Ocean” and is part of an important route to the Atlantic Ocean through the Panama Canal. Pearl Harbor is one of the three major harbors in the Hawaiian archipelago [3], [44]. However, the other Hawaiian island analyzed, Lāna’i, is described as a region of relative isolation with low inter-island commercial traffic and a lack of substantial enclosed harbors [45]. According to the authors, these characteristics seem to be responsible for the lower number of introduced species recorded in Lāna’i in comparison with other locations in the archipelago. Indeed, there were no records in Lāna’i of some common fouling species recorded in the Ilha Grande Bay and Pearl Harbor, such as the bryozoan *B. neritina* and the barnacles of the genus *Amphibalanus*. Despite the relative isolation of Lāna’i, the introduced octocoral *C. riisei* was recorded at this island.

These findings suggest a history of broad-scale invasions that were, likely shipping-mediated, though more evidence is needed to support this hypothesis. Recent advances in molecular techniques may provide useful tools to reconstruct the routes and elucidate the mechanisms of invasion, as previously demonstrated [57]–[59]. Nonetheless, we are aware that the detailed invasion history of the Ilha Grande Bay and other environments, which may include multiple introductions, will never be known with absolute certainty.

Our study is the first to record the introduced bivalve *L. aristata* and the introduced bryozoans *S. diadema* and *S. errata* in the Ilha Grande Bay, which were first recorded in Brazil in 2004 [60], 2002 [61] and 1937 [62], respectively. *S. errata* was recorded at all sites investigated in this study. *L. aristata* was recorded at three of the seven sites sampled, whereas *S. diadema* was recorded at two sites. The introduced species recorded in the present survey, with the exception of *S. diadema*, may be considered established in the Ilha Grande Bay according to a previously proposed classification [7]. This study is also the first report the presence of *A. reticulatus* in natural substrata in this bay. The first observation of this species in the Brazilian coast was recorded in 1988 [63], followed by records on artificial substrata in the Ilha Grande Bay in 1996.

Although the long-distance dispersal vectors of the species introduced to the Ilha Grande Bay remain uncertain, these species were not restricted to the urban areas (Anil Beach, Mombaça and Bracuhy) and artificial substrata of the bay. While a number of researchers, e.g. [12], [64], have emphasized that the prevalence of invasive species may be related to the degree of disturbance in the environment and the availability of human-produced habitats, these findings are still notable, particularly for the recently introduced species *I. bicolor*, *A. reticulatus* and *L. aristata*. In fact, the introduced species recorded in Ilha Grande Bay successfully colonized sites with very different levels of exposure to human disturbances, including harbor, marinas and the islands. It is important to note that these species were not only detected but also were dominant in the zoobenthic community of this ecosystem, as demonstrated by SIMPER analysis.

A similar broad distribution was observed in the different types of substrata. The establishment of *I. bicolor* and its relevant contribution within natural substrata are worth noting. This reef-forming species forms dense beds [65] and potentially acts as a habitat modifier. Ecological studies concerning the interactions between *I. bicolor* and the previously introduced mussel *P. perna* are also critical. Several studies investigating the native species *P. perna*, the associated fauna and the introduced *Mytillus galloprovincialis* have been performed in South Africa. The population of *M. galloprovincialis* has increased in recent years, and it is negatively affecting the native *P. perna* mussel beds and associated fauna. Abiotic components that interact in these associations are also changing [66]–[68].

The Ilha Grande Bay is an environment subjected to recreational boating and intense small-scale fishing and containing several islands and abundant man-made structures. The sum of these factors may provide a reasonable explanation for the establishment and rapid spread of introduced species at the sites analyzed in this study. In fact, the role of recreational boating has been increasingly recognized in the post-border domestic spread of introduced marine species [69]–[75]. Additionally, artificial structures are known to be colonized by non-indigenous epibiota [12], [13], [31]. Although such structures may represent beachheads, *sensu* [64], and appear to be strongly related to bioinvasion events, studies that have specifically tested the habitats used by native versus introduced and cryptogenic species have only recently been conducted [12], [13]. More native species (10%) were recorded on natural substrata at the Ilha Grande Bay, a finding that is consistent with co-evolutionary adaptations between native species and the natural substratum [13]. However, introduced species were similarly recorded on both types of substratum. Despite this finding, distinct patterns of distribution were found for both *I. bicolor* and *S. errata*, the latter of which was previously revealed to be a successful colonizer of artificial substrata [8], [76]. There is no pattern of native and introduced species richness associated with substratum type [12], [13]. Additional studies are required for further elucidation of these associations, which may be strongly influenced by species identity.

If species that colonize artificial structures are able to spread to rocky shores, the proximity of near-shore artificial structures could threaten native rocky shore communities. This may increase the chances that an introduced species can invade and affect native habitats, a possibility that is supported by both classical theories of invasibility [77] and recent experiments [16], [17], [78], [79]. Recent studies have focused on this theme by analyzing introduced species [12], [13], [80]. In the context of the Ilha Grande Bay, several species of bryozoans, ascidians and barnacles were found reproducing on artificial substrata. The distribution patterns of the introduced species *S. errata, I. bicolor* and *B. trigonus* identify them as suitable models for forthcoming investigations of this phenomenon.

The artificial structures analyzed should not be considered adequate surrogates for rocky-shore reefs in the Ilha Grande Bay, as indicated by the observed differences in the diversity and distribution of zoobenthic epibiota. Similar results were found previously [8], [10], [12], [13], [19], [21], [81], [82], though the question of whether man-made structures are good surrogates for natural reefs remains controversial. This question merits attention because shifts in benthic communities may, in theory, exert cascading effects on nektonic and planktonic communities that could result in alterations of the ecosystem's functions.

Our study contributes to the knowledge of invasion along the South Atlantic American coast. An increase in knowledge of the geographic distribution of species in these areas as well as efforts to assess the current zoological inventory are necessary to improve the management of biological invasions and the conservation of natural systems. The introduced species successfully colonized different sites in the Ilha Grande Bay, also including both natural and artificial substrata. The lack of reports on the environmental and economic impacts of these introduced species does not mean that such impacts do not exist, and ecological studies should be considered a priority in this region. Furthermore, our data clearly refute artificial substrata as good surrogates for rocky shores.

## Supporting Information

Table S1 Taxons recorded by this survey at each site and each substratum type (Ilha Grande Bay). *: Probably new species (Fernanda Azevedo, personal communication); **: cryptogenic species; ***: introduced species; ^Δ^: probably a species complex.
(0.23 MB DOC)

Table S2 Species recorded by this survey (Ilha Grande Bay) and their status, origin and the available geographic distributions. C: cryptogenic species; N: native species; I: introduced species.
(0.17 MB DOC)

## References

[1] Ricciardi A (2007). Are modern biological invasions an unprecedented form of global change?. Conserv Biol.

[2] Ruiz GM, Carlton JT, Grosholz ED, Hines AH (1997). Global invasions of marine and estuarine habitats by non-indigenous species: mechanisms, extent and consequences.. Am Zool.

[3] Godwin LS (2003). Hull fouling of maritime vessels as a pathway for marine species invasions to the Hawaiian Islands.. Biofouling.

[4] Bulleri F, Chapman MG (2010). The introduction of coastal infrastructure as a driver of change in marine environments.. J Appl Ecol.

[5] Crisp DJ, Ryland JS (1960). Influences of filming and surface texture on the settlement of marine organisms.. Nature.

[6] McGuinness KA (1989). Effects of some natural and artificial substrata on sessile marine organisms at Galeta Reef, Panama.. Mar Ecol Prog Ser.

[7] Walters LJ, Wethey DS (1996). Settlement and early post settlement survival of sessile marine invertebrates on topographically complex surfaces - the importance of refuge dimensions and adult morphology.. Mar Ecol Prog Ser.

[8] Glasby TM (1999). Differences between subtidal epibiota on pier pilings and rocky reefs at marinas in Sydney, Australia.. Estuar Coast Shelf S.

[9] Glasby TM, Connell SD (1999). Urban structures as marine habitats.. Ambio.

[10] Connell SD (2001). Urban structures as marine habitats: an experimental comparison of the composition and abundance of subtidal epibiota among pilings, pontoons and rocky reefs.. Mar Environ Res.

[11] Bulleri F, Chapman MG (2004). Intertidal assemblages on artificial and natural habitats in marinas on the north-west coast of Italy.. Mar Biol.

[12] Glasby TM, Connell SD, Holloway CL, Hewitt CL (2007). Nonindigenous biota on artificial structures: could habitat creation facilitate biological invasions?. Mar Biol.

[13] Tyrrel MC, Byers JE (2007). Do artificial substrates favor nonindigenous fouling species over native species?. J Exp Mar Biol Ecol.

[14] Sutherland JP, Karlson RH (1977). Development and stability of the fouling community at Beaufort, North Carolina.. Ecol Monogr.

[15] Russ GR (1982). Overgrowth in a marine epifaunal community: competitive hierarchies and competitive networks.. Oecologia.

[16] Stachowicz JJ, Whitlatch RB, Osman RW (1999). Species diversity and invasion resistance in a marine ecosystem.. Science.

[17] Stachowicz JJ, Fried H, Osman RW, Whitlatch RB (2002). Biodiversity, invasion resistance, and marine ecosystem function: reconciling pattern and process.. Ecology.

[18] Butler AJ, Connolly RM (1996). Development and long term dynamics of a fouling assemblage of sessile marine invertebrates.. Biofouling.

[19] Connell SD, Glasby TM (1999). Do urban structures influence local abundance and diversity of subtidal epibiota? A case study from Sydney Harbour.. Mar Environ Res.

[20] Smith SDA, Rule MJ (2002). Artificial substrata in a shallow sublittoral habitat: do they adequately represent natural habitats or the local species pool?. J Exp Mar Biol Ecol.

[21] Bulleri F, Chapman MG, Underwood AJ (2005). Intertidal assemblages on seawalls and vertical rocky shores in Sydney Harbour, Australia.. Austral Ecol.

[22] Creed JC, De Paula AF (2007). Substratum preference during recruitment of two invasive alien corals onto shallow-subtidal tropical rocky shores.. Mar Ecol Progr Ser.

[23] Carlton JT (1996). Biological invasions and cryptogenic species.. Ecology.

[24] Richardson DM, Pyšek P (2008). Fifty years of invasion ecology – the legacy of Charles Elton.. Divers Distrib.

[25] Orensanz JML, Schwindt E, Pastorino G, Bortulos A, Casa G (2002). No longer the pristine confines of the world ocean: a survey of exotic marine species in the southwestern Atlantic.. Biol Invasions.

[26] Neves CS, Rocha RM, Pitombo FB, Roper JJ (2007). Use of artificial substrata by introduced and cryptogenic marine species in Paranaguá Bay, southern Brazil.. Biofouling.

[27] Neves CS, Rocha RM (2008). Introduced and cryptogenic species and their management in Paranaguá Bay, Brazil.. Braz Arch Biol Technol.

[28] Ferreira CEL, Junqueira AOR, Villac MC, Lopes, RM, Rilov G, Crooks JA (2009). Marine bioinvasions in the Brazilian Coast: brief report on history of events, vectors, ecology, impacts and management of non-indigenous species.. Biological invasions in marine ecosystems: ecological, management and geographic perspectives.

[29] Creed JC, Oliveira AES, Pires DO, Figueiredo MAO, Ferreira CEL, Creed JC, Pires DO, Figueriedo MAO (2007). RAP Ilha Grande - um levantamento da biodiversidade: histórico e conhecimento da biota.. Biodiversidade Marinha da Baía da Ilha Grande.

[30] Mayer-Pinto M, Junqueira AOR (2003). Effects of organic pollution on the initial development of fouling communities in a tropical bay, Brazil.. Mar Pollut Bull.

[31] Chapman JW, Carlton JT (1991). A test of criteria for introduced species: the global invasion of the isopod *Synidotea laevidorsalis* (Miers, 1881).. J Crustacean Biol.

[32] Wolda H (1981). Similarity indices, sample size and diversity.. Oecologia.

[33] Lacombe D (1977). Cirripédios da Baía da Ribeira, Angra dos Reis, RJ (Brasil).. Publ Inst Pesq Marine.

[34] Invasive species focus team Gulf of Mexico (2000). inventory of nonindigenous aquatic species occurring in the Gulf of Mexico region: an initial survey of aquatic invasive species issues in the Gulf of Mexico region..

[35] Baker P, Baker SM, Fajans J (2004). Nonindigenous marine species in the greater Tampa Bay ecosystem.. Final Report.

[36] Streftaris N, Zenetos A, Papathanassiou E (2005). Globalization in marine ecosystems: the story of non-indigenous marine species across European seas.. Oceanogr Mar Biol Ann Rev.

[37] Galil BS (2007). Seeing Red: alien species along the Mediterranean coast of Israel.. Aquat Invasions.

[38] Cardigos F, Tempera F, Ávila S, Gonçalves J, Colaço A (2006). Non-indigenous marine species of the Azores.. Res Helgol Mar Res.

[39] Karpinsky MG, Shiganova TA, Katunin DN (2005). Introduced species.. Hdb Env Chem.

[40] Coles SL, Reath PR, Skelton PA, Bonito V, DeFelice RC (2003). Introduced marine species in Pago Pago Harbor, Fagatele Bay and the National Park Coast, American Samoa.. Pacific Biological Survey Contribution No. 2003-007.

[41] Hewitt CL, Campbell ML, Thresher RE, Martin RB, Boyd S (2004). Introduced and cryptogenic species in Port Phillip Bay, Victoria, Australia.. Mar Biol.

[42] Aquenal Pty Ltd. (2001). Exotic marine pests survey Port of Launceston, Tasmania.. Final Report.

[43] Castilla JC, Uribe M, Bahamonde N, Clarke M, Desqueyroux-Faúndez R (2005). Down under the southeastern Pacific: marine non-indigenous species in Chile.. Biol Invasions.

[44] Coles SL, DeFelice RC, Eldredge LG, Carlton JT (1999). Historical and recent introductions of non-indigenous marine species into Pearl Harbor, Oahu, Hawaiian Islands.. Mar Biol.

[45] Coles SL, Longenecker K, Bolick H (2006). Lāna’i nonindigenous marine species survey.. Final Report.

[46] Wasson K, Zabin CJ, Bedinger L, Diaz MC, Pearse JS (2001). Biological invasions of estuaries without international shipping: the importance of intraregional transport.. Biol Conserv.

[47] Pyšek P, Richardson DM, Pergil J, Jarosik V, Sixtova Z (2008). Geographical and taxonomic biases in invasion ecology.. Trends Ecol Evol.

[48] Nuñez MA, Pauchard A (2009). Biological invasions in developing and developed countries: does one model fit all?. Biol Invasions.

[49] Ruiz GM, Fofonoff PW, Carlton JT, Wonhan MJ, Hines AH (2000). Invasion of coastal marine communities in North America: apparent patterns, processes, and biases.. Annu Rev Ecol Syst.

[50] Cohen AN, Harris LH, Bingham BL, Carlton JT, Chapman JW (2005). Rapid assessment survey for exotic organisms in southern California bays and harbors, and abundance in port and non-port areas.. Biol Invasions.

[51] Russell B, Hewitt CL (2000). Baseline survey of the Port of Darwin for introduced marine species.. A report to the Northern Territory Department of Transport and Works Marine Branch, Darwin, Northern Territory, Australia.

[52] Galil BS (2007). Loss or gain? Invasive aliens and biodiversity in the Mediterranean Sea.. Mar Pollut Bull.

[53] Galil BS (2009). Taking stock: inventory of alien species in the Mediterranean.. Biol Invasions.

[54] Carlton JT (1989). Man's role in changing the face of the ocean: biological invasions and implications for conservation of nearshore environments.. Conserv Biol.

[55] Geller JB, Darling JA, Carlton JT (2010). Genetic perspectives on marine biological invasions.. Annu Rev Mar Sci.

[56] Klautau M, Russo CAM, Lazoski C, Boury-Esnault N, Thorpe JP (1999). Does cosmopolitanism result from overconservative systematics? A case study using the marine sponge *Chondrilla nucula*.. Evolution.

[57] Audzijonyte A, Wittmann KJ, Väinölä R (2008). Tracing recent invasions of the Ponto-Caspian mysid shrimp *Hemimysis anomal* across Europe and to North America with mitochondrial DNA.. Divers Distrib.

[58] Hufbauer RA, Sforza R (2008). Multiple introductions of two invasive *Centaurea taxa* inferred from cpDNA haplotypes.. Divers Distrib.

[59] Provan J, Booth D, Todd NP, Beatty GE, Maggs CA (2008). Tracking biological invasions in space and time: elucidating the invasive history of the green alga *Codium fragile* using old DNA.. Divers Distrib.

[60] Simone LRL, Gonçalves EP (2006). Anatomical study on *Myoforceps aristatus*, an invasive boring bivalve in S.E. Brazilian coast (Mytilidae).. Pap Avulsos Zool.

[61] Ramalho LV (2006). Taxonomia, distribuição e introdução de espécies de briozoários marinhos (Ordens Cheilostomatida e Cyclostomata) do estado do Rio de Janeiro..

[62] Vieira LM, Migotto AE, Winston JE (2008). Synopsis and annotated checklist of recent marine Bryozoa from Brazil.. Zootaxa.

[63] Farrapeira CMR (2008). Cirripedia Balanomorpha del estuario del Río Paripe (Isla de Itamaracá, Pernambuco, Brasil).. Biota Neotrop.

[64] Simberloff D, Simberloff D, Schmitz DC, Brow TC (1997). The biology of invasions.. Strangers in paradise: impact and management of nonindigenous species in Florida.

[65] Breves-Ramos A, Junqueira AOR, Lavrado HP, Silva SHG, Ferreira-Silva MAG (2010). Population structure of the invasive bivalve *Isognomon bicolor* on rocky shores of Rio de Janeiro State (Brazil).. J Mar Biol Assoc UK.

[66] Nicastro KR, Zardi GI, McQuaid CD (2007). Behavioural response of invasive *Mytilus galloprovincialis* and indigenous *Perna perna* mussels exposed to risk of predation.. Mar Ecol Prog Ser.

[67] Hanekom N (2008). Invasion of an indigenous *Perna perna* mussel bed on the south coast of South Africa by an alien mussel *Mytilus galloprovincialis* and its effect on the associated fauna.. Biol Invasions.

[68] Zardi GI, Nicastro KR, McQuaid CD, Erlandsson J (2008). Sand and wave induced mortality in invasive (*Mytilus galloprovincialis*) and indigenous (*Perna perna*) mussels.. Mar Biol.

[69] Fletcher RL, Farrell P (1998). Introduced brown algae in the North East Atlantic, with particular respect to *Undaria pinnatifida* (Harvey) Suringar.. Helgol Meersunters.

[70] Lambert CC, Lambert G (1998). Non-indigenous ascidians in southern California harbors and marinas.. Mar Biol.

[71] Hutchings PA, Hilliard RW, Coles SL (2002). Species introduction and potential for marine pest invasions into tropical marine communities, with special reference to the Indo-Pacific.. Pac Sci.

[72] Floerl O, Inglis GJ (2003). Boat harbour design can exacerbate hull fouling.. Aust Ecol.

[73] Floerl O, Inglis GJ, Marsh HM (2005). Selectivity in vector management: an investigation of the effectiveness of measures used to prevent transport of non-indigenous species.. Biol Invasions.

[74] Wyatt ASJ, Hewitt CL, Walker DI, Ward TJ (2005). Marine introductions in the Shark Bay World Heritage Property, Western Australia: a preliminary assessment.. Divers Distrib.

[75] Ashton G, Boos K, Shucksmith R, Cook E (2006). Risk assessment of hull fouling as a vector for marine non-natives in Scotland.. Aquat Inv.

[76] Floerl O, Pool TK, Inglis GJ (2004). Positive interactions between nonindigenous species facilitate transport by human vectors.. Ecol Appl.

[77] Elton CS (1958). The ecology of invasions by animals and plants..

[78] Clark GF, Johnston EL (2005). Manipulating larval supply in the field: a controlled study of marine invasibility.. Mar Ecol Prog Ser.

[79] Stachowicz JJ, Byrnes JE (2006). Species diversity, invasion success, and ecosystem functioning: disentangling the influence of resource competition, facilitation, and extrinsic factors.. Mar Ecol Prog Ser.

[80] Kremer LP, Rocha RM, Roper JJ (2010). An experimental test of colonization ability in the potentially invasive *Didemnum perlucidum* (Tunicata, Ascidiacea).. Biol Invasions.

[81] Bacchiocchi F, Airoldi L (2003). Distribution and dynamics of epibiota on hard structures for coastal protection.. Estuar Coast Shelf S.

[82] Vaselli S, Bulleri F, Benedetti-Cecchi L (2008). Hard coastal-defense structures as habitats for native and exotic rocky-bottom species.. Mar Environ Res.

