# Supplementary material for: Bioinvasion in a Brazilian Bay: Filling Gaps in the Knowledge of Southwestern Atlantic Biota
Source: PLoS One. 2010 Sep 29;5(9):e13065. doi: 10.1371/journal.pone.0013065 (PMC2947507; doi:10.1371/journal.pone.0013065)
Supplement: Table S1 — Taxons recorded by this survey at each site and each substratum type (Ilha Grande Bay). *: Probably new species (Fernanda Azevedo, personal communication); **: cryptogenic species; ***: introduced species; Δ: probably a species complex. (0.23 MB DOC) [file pone.0013065.s001.doc]

|  |  | **natural** | | | | | **artificial** | | | | |
| --- | --- | --- | --- | --- | --- | --- | --- | --- | --- | --- | --- |
|  |  | **G** | **I1** | **I2** | **I3** | **A** | **G** | **I3** | **B** | **M** | **A** |
| **Porifera** | *Amorphinops* sp. |  |  |  |  |  |  |  |  |  | X |
|  | *Amphimedon viridis* | X | X |  | X |  |  |  |  |  |  |
|  | *Clathrina* sp. * |  |  |  |  |  |  |  |  |  | X |
|  | *Desmapsamma anchorata* |  |  |  |  |  | X |  |  |  |  |
|  | *Dysidex* cf. *janiae* | X |  |  |  |  |  |  |  |  |  |
|  | *Geodia* sp. |  |  |  |  |  |  |  |  |  | X |
|  | Halichondrida 1 |  |  |  |  |  |  |  |  | X | X |
|  | Haplosclerida 1 | X | X | X | X | X |  |  |  | X | X |
|  | *Leucandra* *serrata* |  |  |  |  |  |  |  |  |  | X |
|  | *Leucandra* sp. 1 |  |  |  |  |  |  |  |  | X | X |
|  | *Leucandra* sp. 2 |  |  |  |  |  |  |  |  |  | X |
|  | *Lissodendoryx isodictyalis* **^△^ |  |  |  |  |  |  |  |  | X | X |
|  | *Mycale aff. magnirhaphidifera* |  |  |  |  |  |  |  |  |  | X |
|  | *Mycale angulosa* |  |  |  |  |  | X | X | X | X | X |
|  | *Mycale microsigmatosa* |  |  |  |  | X | X | X | X | X | X |
|  | *Stelletta* sp. |  |  |  |  |  |  |  |  |  | X |
|  | *Sycon* sp. |  |  |  |  |  |  |  |  | X |  |
|  | *Tedania ignis* | X | X | X | X | X | X | X |  | X | X |
|  | *Terpios* sp. | X |  |  |  |  |  |  |  |  |  |
|  | *Tethya* sp. |  |  |  |  | X |  |  |  |  |  |
| **Cnidaria - Anthozoa** | *Carijoa riisei**** |  | X |  |  |  |  | X |  | X | X |
|  | *Leptogorgia punicea* |  | X |  |  |  |  |  |  |  |  |
|  | *Palythoa caribaeorum* | X | X | X | X |  |  |  |  |  |  |
| **Bryozoa** | *Aetea anguina* ** |  |  | X |  |  |  |  |  |  |  |
|  | *Aetea ligulata* ** |  |  |  |  |  |  |  |  |  | X |
|  | *Aetea truncata* ** |  |  |  |  |  |  |  |  |  | X |
|  | *Amathia* sp. 1 |  | X | X |  |  |  |  |  | X | X |
|  | *Amathia* sp. 2 |  |  |  |  |  |  |  |  | X | X |
|  | *Beania cupulariensis* ** |  |  | X |  |  |  |  |  |  |  |
|  | *Beania klugei* ** |  |  | X |  |  |  |  |  |  | X |
|  | *Beania mirabilis* ** |  |  | X |  |  |  |  |  |  | X |
|  | *Bicrisia* sp. |  |  |  |  |  |  |  |  |  | X |
|  | *Bugula carvalhoi* |  |  |  |  |  |  |  |  |  | X |
|  | *Bugula neritina ***^△^ |  |  |  |  | X |  |  |  |  | X |
|  | *Bugula uniserialis *** |  |  | X |  |  |  |  |  |  |  |
|  | *Catenicella uberrima *** |  |  | X |  |  |  |  |  | X |  |
|  | *Cellaria* sp. 1 |  |  | X |  |  |  |  |  |  |  |
|  | *Celleporaria* sp. 1 |  |  |  |  |  |  |  |  |  | X |

|  |  | **natural** | | | | | **artificial** | | | | |
| --- | --- | --- | --- | --- | --- | --- | --- | --- | --- | --- | --- |
|  |  | **G** | **I1** | **I2** | **I3** | **A** | **G** | **I3** | **B** | **M** | **A** |
| **Bryozoa (continued)** | *Crisia* sp. |  |  |  |  |  |  |  |  |  | X |
|  | Crisiidae 1 |  |  |  |  |  |  |  |  |  | X |
|  | *Exidmonea* sp. 1 |  | X |  |  |  |  |  |  |  |  |
|  | *Savignyella lafontii *** |  |  | X |  |  |  | X |  | X | X |
|  | *Schizoporella errata **** |  | X | X |  | X | X | X | X | X | X |
|  | *Scrupocellaria* aff. *reptans* |  |  |  |  | X |  |  |  | X | X |
|  | *Scrupocellaria cornigera *** |  |  |  |  |  |  |  |  |  | X |
|  | *Scrupocellaria diadema **** |  | X | X |  |  |  |  |  |  |  |
|  | *Synnotum aegyptiacum *** |  |  |  |  |  |  |  |  |  | X |
| **Mollusca** | *Arca imbricata* | X | X |  |  |  |  |  |  |  | X |
|  | *Brachidontes exustus* |  | X | X |  |  |  |  |  |  | X |
|  | *Chama (Pseudochama) radians* | X | X | X | X | X | X | X |  | X |  |
|  | *Chama* sp. | X | X | X | X |  |  |  |  | X | X |
|  | *Choristodon robustus* |  | X |  |  | X |  |  |  |  | X |
|  | *Crassinella lunulata* |  | X |  |  |  |  |  |  |  |  |
|  | *Crassostrea rhizophorae* | X | X | X |  | X |  | X | X | X | X |
|  | *Globivenus (Ventricolaria) rigida* |  | X |  |  |  |  |  |  |  |  |
|  | *Gouldia cerina* |  | X |  |  |  |  | X |  |  |  |
|  | *Hiatella arctica ***^△^ | X | X | X |  | X |  | X |  |  | X |
|  | *Isognomon bicolor **** | X | X | X | X | X | X | X |  | X | X |
|  | *Lectopecten bavayi* |  |  | X |  |  |  |  |  |  |  |
|  | *Lithophaga bisulcata* |  | X | X |  |  |  | X |  |  |  |
|  | *Lithophaga (Myoforceps) aristata **** | X | X | X |  |  |  |  |  |  | X |
|  | *Modiolus carvalhoi* |  | X | X |  | X | X | X |  | X | X |
|  | *Musculus lateralis* |  | X | X |  | X |  |  |  |  | X |
|  | *Mytella charruana* |  | X |  |  |  |  |  |  |  |  |
|  | *Perna perna **** |  |  | X |  |  |  |  |  |  | X |
|  | *Petaloconchus varians* | X | X | X | X | X | X | X |  |  | X |
|  | *Pinctada imbricata* | X | X | X |  |  |  |  |  |  | X |
| **Crustacea - Cirripedia** | *Amphibalanus eburneus *** |  |  |  |  |  |  | X | X |  |  |
|  | *Amphibalanus improvisus *** |  |  |  |  | X |  | X | X |  |  |
|  | *Amphibalanus reticulatus **** |  |  |  | X | X | X | X | X |  |  |
|  | *Balanus trigonus **** | X | X | X | X | X | X | X | X | X | X |
|  | *Megabalanus coccopoma **** |  |  |  |  |  | X |  |  |  |  |
|  | *Newmanella radiata* | X |  |  |  |  | X |  |  |  |  |
| **Chordata - Ascidiacea** | *Botrylloides nigrum *** |  |  |  |  | X |  |  | X |  | X |
|  | *Clavelina oblonga *** |  |  |  |  |  |  |  |  |  | X |
|  | Didemnidae 1 | X |  | X |  | X |  |  | X | X |  |
|  | *Diplosoma listerianum ***^△^ |  |  | X |  | X |  |  | X |  |  |
|  | *Distaplia bermudensis *** |  |  |  |  |  |  |  | X |  | X |

|  |  | **natural** | | | | | **artificial** | | | | |
| --- | --- | --- | --- | --- | --- | --- | --- | --- | --- | --- | --- |
|  |  | **G** | **I1** | **I2** | **I3** | **A** | **G** | **I3** | **B** | **M** | **A** |
| **Chordata – Ascidiacea** | *Herdmania pallida *** | X | X | X | X | X | X |  |  | X | X |
| **(continnued)** | *Lissoclinum* sp. | X |  |  |  |  |  |  |  |  |  |
|  | *Microcosmus exasperatus *** | X | X |  |  | X |  |  | X |  | X |
|  | *Phallusia nigra *** | X |  |  |  |  |  | X |  | X |  |
|  | *Styela canopus *** |  |  |  |  |  |  |  | X |  |  |
|  | *Styela plicata **** |  |  |  |  | X |  |  |  |  | X |
|  | *Symplegma rubra *** |  |  |  |  |  |  |  |  | X |  |
