# Supplementary material for: Bioinvasion in a Brazilian Bay: Filling Gaps in the Knowledge of Southwestern Atlantic Biota
Source: PLoS One. 2010 Sep 29;5(9):e13065. doi: 10.1371/journal.pone.0013065 (PMC2947507; doi:10.1371/journal.pone.0013065)
Supplement: Table S2 — Species recorded by this survey (Ilha Grande Bay) and their status, origin and the available geographic distributions. C: cryptogenic species; N: native species; I: introduced species. (0.17 MB DOC) [file pone.0013065.s002.doc]

|  | **Taxon** | **Status** | **Origin and Distribution** |
| --- | --- | --- | --- |
| **Porifera** | *Amphimedon viridis* | N | **Current distribution:** United States of America (North Carolina and Florida); Caribbean (Saint Thomas, Curaçao, Puerto Rico, Bahamas and Bermuda) and Brazil (Pernambuco, Alagoas, Bahia, Rio de Janeiro and São Paulo) (Muricy & Hajdu, 2006) |
|  | *Clathrina* sp. * | N | **Original and Current distributions**: Original and endemic from Brazil: Rio de Janeiro |
|  | *Desmapsamma anchorata* | N | **Current distribution**: Tropical Atlantic: Caribbean and Brazil (Pernambuco, Bahia and Rio de Janeiro) (Muricy & Hajdu, 2006) |
|  | *Leucandra* *serrata* | N | **Original and Current distributions:** Brazil: Rio de Janeiro (Azevedo & Klautau, 2007). |
|  | *Lissodendoryx isodictyalis* ^Δ^ | C | **Current distribution**: United States of America (North Carolina), Mexico Pacific coast, Caribbean, Mediterranean Sea. Indian (Smithsonian Tropical Research Institute– www.si.edu), Atlantic and Pacific oceans (Hewitt *et al*., 2004) |
|  | *Mycale angulosa* | N | **Current distribution**: Brazil: Pernambuco, Bahia, Rio de Janeiro and São Paulo (Muricy & Hajdu, 2006) |
|  | *Mycale microsigmatosa* | N | **Current distribution**: United States of America (Florida), Caribbean (Curaçao, Bonaire, Isla Margarita, Colombia, Jamaica, Panama, Belize) and Brazil (Bahia, Rio de Janeiro, São Paulo and Santa Catarina) (Muricy & Hajdu, 2006) |
|  | *Tedania ignis* | N | **Current distribution**: Widely distributed in Southwest Atlantic and Caribbean. Brazil: Piauí to Santa Catarina (Muricy & Hajdu, 2006) |
| **Cnidaria - Anthozoa** | *Carijoa riisei* | I | **Original distribution:** Indo-Pacific (Concepcion *et al*., 2010). **Current distribution**: Western Atlantic (United States of America (Florida) to Brazil), Eastern Atlantic (Sierra Leone), Indian Ocean (Zanzibar), Indo-Pacific (Sion Gulf, Singapore, Shanghai, Sumatra, Manila, “New Britain”, Hawaii). Brazil: Amapá to Santa Catarina, St Paul´s Rocks and Vitoria Seamount (Castro *et al.,* 2010) |
|  | *Leptogorgia punicea* | N | **Current distribution**: East coast of America (United States of America (Florida) to Brazil). Brazil: Maranhão, Bahia to Rio Grande do Sul (Castro *et al.,* 2010) |
|  | *Palythoa* *caribaeorum* | N | **Current distribution:** Uncertain. Brazilian coast is considered as part of the original distribution. |
| **Bryozoa** | *Aetea anguina* | C | **Current distribution**: Cosmopolitan distribution. Brazil: Pernambuco, Rio de Janeiro and São Paulo (Ramalho, 2006) |
|  | *Aetea ligulata* | C | **Current distribution**: Widely distributed. South Atlantic and Pacific Ocean (Australia, British Columbia, United States of America-California and Colombia). Brazil: Rio de Janeiro and São Paulo (Ramalho, 2006) |
|  | *Aetea truncata* | C | **Current distribution**: Widely distributed. Western Atlantic, Indian and Pacific oceans. Brazil: Rio de Janeiro and São Paulo (Ramalho, 2006) |
|  | *Beania cupulariensis* | C | **Current distribution**: Caribbean, Brazil and Africa Western coast (Cook, 1985) |
|  | *Beania klugei* | C | **Current distribution**: Northwest and Southwest Atlantic, Southwest Pacific and Indian, Red Sea. Brazil: Pernambuco, Rio de Janeiro and São Paulo (Ramalho, 2006) |
|  | *Beania mirabilis* | C | **Current distribution**: North and South Atlantic, Mediterranean Sea, Northeast e South Indian and Northeast Pacific. Brazil: Rio de Janeiro and São Paulo (Ramalho, 2006) |
|  | *Bugula carvalhoi* | N | **Original and Current distributions**: Original and endemic from Brazil (Marcus, 1949). Brazil: Rio de Janeiro and São Paulo (Ramalho, 2006) |
|  | *Bugula neritina* ^Δ^ | C | **Current distribution**: Cosmopolitan in tropical regions. Brazil: Rio de Janeiro, São Paulo and Paraná (Ramalho, 2006) |
|  | *Bugula uniserialis* | C | **Current distribution**: Eastern Pacific (California and Galapagos), Eastern Indian (Australia), Western Atlantic (United States of America-Florida and Brazil). Brazil: Rio de Janeiro and São Paulo (Ramalho, 2006) |
| *** probably new species (Fernanda Azevedo, personal communications); ^Δ^** **probably a species complex.** | | | |

|  | **Taxon** | **Status** | **Origin and Distribution** |
| --- | --- | --- | --- |
| **Bryozoa (continued)** | *Catenicella uberrima* | C | **Current distribution**: Western Atlantic (Bermuda, United States of America-Florida, Gulf of Mexico, Caribbean, Brazil), Eastern Atlantic (African coast), Pacific Ocean (Singapore and New Guinea). Brazil: Rio de Janeiro (Ramalho, 2006) |
|  | *Savignyella lafontii* | C | **Current distribution**: Widely distributed in tropical seas. Western Atlantic (Bermudas, United States of America-Florida, Gulf of Mexico to Brazil), Indian Ocean, Red and Mediterranean seas. Brazil: Rio de Janeiro and São Paulo (Ramalho, 2006) |
|  | *Schizoporella errata* | I | **Current distribution**: Widely distributed in warm, temperate and subtropical seas. Eastern and Western Atlantic, Western Pacific, Red and Mediterranean seas. Brazil: Rio de Janeiro and São Paulo (Ramalho, 2006; Vieira *et al.,* 2008; Tompsett *et al.,* 2009) |
|  | *Scrupocellaria cornigera* | C | **Current distribution**: Atlantic Ocean (United States of America-Florida and Brazil) and Pacific Ocean (Tobago). Brazil: Rio de Janeiro (Ramalho, 2006) |
|  | *Scrupocellaria diadema* | I | **Original distribution:** Indo-Pacific. **Current distribution**: Indo-Pacific and Brazil (Rio de Janeiro) (Ramalho, 2006) |
|  | *Synnotum aegyptiacum* | C | **Current distribution**: Widely distributed in tropical seas. Atlantic, Indian and Pacific oceans; Red and Mediterranean seas. Brazil: Fernando de Noronha, Espírito Santo, Rio de Janeiro and São Paulo. (Ramalho, 2006) |
| **Mollusca** | *Arca imbricata* ^❒^ | N | **Current distribution**: Bermuda, Bahamas, Gulf of Mexico, Caribbean Central America to Brazil (Mikkelsen & Bieler, 2008). Brazil: Pará to Santa Catarina (Rios, 1994) |
|  | *Brachidontes exustus* ^❒^ | N | **Current distribution**: United States of America (New Jersey to Florida), Western Indies, Bermuda, Bahamas, Western Indies, Gulf of Mexico, Caribbean Central America, Brazil to Argentina, Saint Helena (Mikkelsen & Bieler, 2008) |
|  | *Chama (Pseudochama) radians* ^❒^ | N | **Current distribution**: United States of America (North Carolina to Florida), Bermuda, Bahamas, Gulf of Mexico, Western Indies, Caribbean Central America, South America to Brazil, Western Europe (Mikkelsen & Bieler, 2008). Brazil: Maranhão to Santa Catarina (Rios, 1994) |
|  | *Choristodon robustus* ^❒^ | N | **Current distribution**: United States of America (North Carolina to Florida), Bermuda, Bahamas, Gulf of Mexico, Western Indies, Caribbean Central America, South America to Brazil, Eastern Pacific (Mikkelsen & Bieler, 2008). Brazil: (Fernando de Noronha, Rio Grande do Norte to Santa Catarina) (Rios, 1994) |
|  | *Crassinella lunulata* | N | **Current distribution**: United States of America (Massachusetts to Florida), Bermuda, Bahamas, Western Indies, Gulf of Mexico, Caribbean Central America, South America to Uruguay (Mikkelsen & Bieler, 2008) Brazil: Amapá to Santa Catarina (Rios, 1994) |
|  | *Crassostrea rhizophorae* ^❒^ | N | **Current distribution**: United States of America (Florida Keys), Western Indies, Gulf of Mexico, Caribbean Central America, South America to Uruguay (Mikkelsen & Bieler, 2008) |
|  | *Globivenus (Ventricolaria rigida)* ^❒^ | N | **Current distribution**: United States of America (North Carolina to Florida), Bahamas, Western Indies, Gulf of Mexico, Caribbean Central America, South America to Brazil (Mikkelsen & Bieler, 2008). Brazil: Rio Grande do Norte to Santa Catarina (Rios, 1994) |
|  | *Gouldia cerina* | N | **Current distribution**: United States of America (Maryland to Florida), Bermuda, Bahamas, Western Indies, Gulf of Mexico, Caribbean Central America, South America to Brazil (Mikkelsen & Bieler, 2008). Brazil: Amapá to Rio Grande do Sul (Rios, 1994) |
|  | *Hiatella arctica* ^Δ^ | C | **Current distribution**: North Atlantic to Argentina, Eastern Pacific, Western Europe, Antarctic Island (Mikkelsen & Bieler, 2008) |
|  |  |  |  |

**^Δ^** **probably a species complex; ^❒^species found in shellmound from Rio de Janeiro, Brazil (Souza *et al,* 2010).**

|  | **Taxon** | **Status** | **Origin and Distribution** |
| --- | --- | --- | --- |
| **Mollusca (continued)** | *Isognomon bicolor* | I | **Original distribution:** Caribbean (Domaneschi & Martins, 2002). **Current distribution**: United States of America (Florida), Bermuda, Bahamas, Western Indies, Gulf of Mexico, Caribbean Central America, Colombia to Brazil (Mikkelsen & Bieler, 2008). Brazil: Rio Grande do Norte, Pernambuco, Bahia, Rio de Janeiro, São Paulo and Santa Catarina (Domaneschi & Martins, 2002) |
|  | *Lectopecten bavayi* | N | **Current distribution**: Western Indies, Colombia, Venezuela, Brazil and Uruguay (Rios, 1994) |
|  | *Lithophaga bisulcata* | N | **Current distribution**: United States of America (North Carolina to Florida), Bermuda, Bahamas, Western Indies, Gulf of Mexico, Caribbean Central America, South America to Uruguay (Mikkelsen & Bieler, 2008). Brazil: Maranhão to São Paulo and Trindade (Rios, 1994) |
|  | *Lithophaga (Myoforceps) aristata* | I | **Original distribution:** Caribbean (Simone & Gonçalves, 2006). **Current distribution**: Cosmopolitan. North Atlantic (Portugal to Senegal, United States of America-North Carolina to Venezuela), Pacific coast of South America, Red Sea, Australia, Japan (Abbott, 1974) and Brazil (Rio de Janeiro and São Paulo) (Simone & Gonçalves, 2006, Mikkelsen & Bieler, 2008) |
|  | *Modiolus carvalhoi* | N | **Current distribution**: Brazil: Rio Grande do Norte, Rio de Janeiro to Rio Grande do Sul (Rios, 1994; Silveira *et al*., 2006) |
|  | *Musculus lateralis* | N | **Current distribution**: United States of America (North Carolina to Florida), Bermuda, Bahamas, Western Indies, Gulf of Mexico, Caribbean Central America, Colombia, Venezuela and Brazil (Mikkelsen & Bieler, 2008). Brazil: Pernambuco to Santa Catarina and Trindade (Rios, 1994). |
|  | *Mytella charruana* ^❒^ | N | **Current distribution**: Mexico to Guiana, Ecuador, Galapagos, Venezuela, Suriname, Brazil, Uruguay and Argentina (Rios, 1994) |
|  | *Perna perna* | I | **Original distribution:** Eastern Atlantic - Africa (possibly Congo, Angola, Mozambique and Tanzania) (Souza *et. al.,* 2003)**. Current distribution**: Tropical and subtropical regions of Atlantic and Indian oceans and Mediterranean Sea. Brazil: Rio Grande do Norte, Espírito Santo to Rio Grande do Sul (Souza *et al*., 2003; Silveira *et al*., 2006) |
|  | *Petaloconchus varians* | N | **Current distribution**: Western Atlantic. Brazil: Ceará, Fernando de Noronha, Pernambuco, Espírito Santo, Rio de Janeiro, São Paulo and Santa Catarina (Rios, 1994) |
|  | *Pinctada imbricata* ^❒^ | N | **Current distribution**: United States of America (North Carolina to Florida), Bermuda, Bahamas, Gulf of Mexico, Western Indies, Caribbean Central America, South America to Brazil (Mikkelsen & Bieler, 2008). Brazil: Pará to Santa Catarina (Rios, 1994) |
| **Crustacea - Cirripedia** | *Amphibalanus eburneus* | C | **Original distribution:** Northwestern Atlantic Ocean (uncertain about the Southwestern Atlantic Ocean) (Zullo, 1992). **Current distribution**: Widely distributed in tropical seas (Young, 1998) |
|  | *Amphibalanus improvisus* | C | **Original distribution:** North Atlantic Ocean (uncertain if eastern, western or both South Atlantic Ocean) (Zullo, 1992). **Current distribution**: Widely distributed. New Scotland to Patagonia, Scotland, Baltic Sea to African West coast, Mediterranean Sea, Black Sea, Caspian Sea, Red Sea, Japan, Australia, United States of America (Oregon) to Peru and Brazil (Ceará to Rio Grande do Sul) (Young, 1994) |
|  | *Amphibalanus reticulatus* | I | **Original distribution:** Eastern Pacific (Japan) (Utinomi, 1967). **Current distribution**: Widely distributed in Circumtropical regions. Brazil: Maranhão, Alagoas, Pernambuco, Bahia, Rio de Janeiro and Paraná (Young, 1998) |
|  | *Balanus trigonus* | I | **Original distribution:** Pacific Ocean (Zullo, 1992). **Current distribution**: Widely distributed. Brazil: Amapá to Rio Grande do Sul (Young, 1998) |
|  |  |  |  |

**^Δ^** **probably a species complex; ^❒^species found in shellmound from Rio de Janeiro, Brazil (Souza *et al,* 2010).**

|  | **Taxon** | **Status** | **Origin and Distribution** |
| --- | --- | --- | --- |
|  | *Megabalanus coccopoma* | I | **Original distribution:** Eastern Pacific (United States of America-California to Peru). **Current distribution**: Pacific Ocean (United States of America to Peru, Galapagos and New Caledonia), Indian Ocean (Mauritius Island) and Atlantic Ocean (Brazil). Brazil: Rio Grande do Norte, Espírito Santo to Rio Grande do Sul (Young, 1998; Silveira *et al*., 2006) |
|  | *Newmanella radiata* | N | **Current distribution**: Western Atlantic. Brazil: Pernambuco, Rio de Janeiro and São Paulo (Young, 1998) |
| **Chordata - Ascidiacea** | *Botrylloides nigrum* | C | **Current distribution**: Atlantic, Pacific and Indian oceans. Brazil: Pernambuco, Alagoas, Bahia to Santa Catarina (Marins *et al.,* 2010) |
|  | *Clavelina oblonga* | C | **Current distribution**: Western and Eastern Atlantic. Brazil: Espírito Santo to Santa Catarina (Marins *et al.,* 2010) |
|  | *Diplosoma listerianum* ^Δ^ | C | **Current distribution**: Atlantic, Pacific and Indian oceans. Brazil: Rio Grande do Norte, Pernambuco, Alagoas, Bahia to Santa Catarina (Marins *et al,.* 2010) |
|  | *Distaplia bermudensis* | C | **Current distribution**: American Tropical Atlantic (Rodrigues *et al.,* 1998) and African Atlantic (Pérès, 1949). Brazil: Pará, Espírito Santo to Santa Catarina (Rocha & Kremer, 2005) |
|  | *Herdmania pallida* | C | **Current distribution**: Atlantic, Pacific and Indian oceans. Brazil: Ceará, Alagoas, Bahia, Rio de Janeiro and São Paulo (Marins *et al.,* 2010) |
|  | *Microcosmus exasperatus* | C | **Current distribution**: Atlantic, Pacific and Indian oceans and Mediterranean Sea. Brazil: Ceará, Pernambuco, Alagoas, Bahia, Rio de Janeiro to Santa Catarina (Marins *et al.,* 2010) |
|  | *Phallusia nigra* | C | **Current distribution**: Atlantic, Pacific and Indian oceans, Mediterranean and Red seas. Brazil: Ceará, Alagoas, Bahia, Rio de Janeiro and São Paulo (Marins *et al.,* 2010) |
|  | *Styela canopus* | C | **Current distribution**: Atlantic, Pacific and Indian oceans. Brazil: Rio Grande do Norte, Pernambuco, Bahia, Rio de Janeiro to Santa Catarina (Marins *et al.,* 2010) |
|  | *Styela plicata* | I | **Original distribution:** Northwestern Pacific (Kott, 1998 *apud* Carlton, 2009). **Current distribution**: Atlantic, Pacific and Indian oceans and Mediterranean Sea. Brazil: Bahia, Rio de Janeiro to Santa Catarina (Marins *et al.* 2010) |
|  | *Symplegma rubra* | C | **Current distribution**: Western Atlantic, Pacific and Indian oceans. Brazil: Espírito Santo to Paraná (Marins *et al.,* 2010) |

**^Δ^** **probably a species complex.**

**References:**

Abbott RT (1974) American Seashells. Second edition. New York: Van Nostrand Reinhold Company. 663p+240pls.

Azevedo F, Klautau M (2007) Calcareous sponges (Porifera, Calcarea) from Ilha Grande Bay, Brazil, with descriptions of three new species. Zootaxa 1402: 1–22.

Carlton JT (2009) Deep invasion ecology and the assembly of communities in historical time, In: Rilov G, Crooks JA, editors. Biological invasions in marine ecosystems: ecological, management and geographic perspectives. Berlin: Springer-Verlag, pp. 13-56.

Castro CB, Medeiros MS, Loiola LL (2010) Octocorallia (Cnidaria: Anthozoa) from Brazilian reefs. J Nat Hist 44 (13-14): 763–827.

Concepcion GT, Kahng SE, Crepeau MW, Franklin EC, Coles SL, et al. (2010) Resolving natural ranges and marine invasions in a globally distributed octocoral (genus *Carijoa*). Mar Ecol Prog Ser 401: 113–127.

Cook PL (1985) Bryozoa from Ghana, a preliminary survey. Zool. Wetenschappen, 238. Tervuren (Belgique): Musée Royal de l.Afrique Centrale. 315p.

Domaneschi O, Martins CM (2002) *Isognomon bicolor* (C.B. Adams) (Bivalvia, Isognomonidae): primeiro registro para o Brasil, redescrição da espécie e considerações sobre a ocorrência e distribuição de *Isognomon* na costa brasileira. Rev Bras Zool 19 (2): 611-627.

Hewitt CL, Campbell ML, Thresher RE, Martin RB, Boyd S, et al. (2004) Introduced and cryptogenic species in Port Phillip Bay, Victoria, Australia. Mar Biol 144: 183–202.

Marcus E (1949) Some Bryozoa from Brazilian coast. Comm Zool Mus Hist Nat Montevideo 3(53): 1-33.

Marins FO, Novaes RLM, Rocha RM, Junqueira AOR (2010) Non indigenous ascidian in port and natural environment in a tropical Brazilian bay. Zoologia 27 (2): 213-221.

Mikkelsen P, Bieler R (2008) Seashells of Southern Florida. Living marine mollusks of the Florida keys and adjacent regions. Princeton and Oxford: Princeton University Press. 503p.

Muricy G, Hajdu E (2006) Porifera Brasilis: guia de identificação das esponjas marinhas do sudeste do Brasil. Museu Nacional Série Livros 17: Eclesiarte Press. 104p.

Pérès JM (1949) Contribution à l’étude des Ascidies de la côte occidentale d’Afrique. Bull. Inst Fr Afrique Noire: 11(1-2): 159-207.

Ramalho LV (2006) Taxonomia, distribuição e introdução de espécies de briozoários marinhos (Ordens Cheilostomatida e Cyclostomata) do estado do Rio de Janeiro. Rio de Janeiro, Brazil: Universidade Federal do Rio de Janeiro. 450p.

Rios EC (1994) Seashells of Brazil. Rio Grande: FURG Press. 492p.

Rocha RM, Kremer LP (2005) Introduced Ascidians in Paranaguá Bay, Paraná, Southern Brazil. Rev Bras Zool 22(4): 1170-1184.

Rodrigues SA, Rocha RM, Lotufo TMC (1998) Guia ilustrado para identificação das ascídias do estado de São Paulo. São Paulo: IB USP/FAPESP Press. 190 p.

Silveira NG, Souza RCCL, Fernandes FC, Silva EP (2006) Occurrence of *Perna perna*, *Modiolus carvalhoi* (Mollusca, Bivalvia, Mytillidae) and *Megabalanus coccopoma* (Crustacea, Cirripedia) of Areia Branca, Rio Grande do Norte state, Brazil. Biociências 14(1): 89-90.

Simone LRL, Gonçalves EP (2006) Anatomical study on *Myoforceps aristatus*, an invasive boring bivalve in S.E. Brazilian coast (Mytilidae). Pap Avulsos Zool 46 (6): 57-65.

Souza RCCL, Fernandes FC, Silva EPA (2003) A study on the occurrence of the brown mussel *Perna perna* on the sambaquis of the Brazilian coast. Rev Museu de Arqueologia e Etnologia 13: 3-24.

Souza RCCL, Lima TA, Silva EP (2010) Holocene molluscs from Rio de Janeiro state coast, Brazil. Check List 6 (2): 301-308.

Tompsett S, Porter JS, Taylor PD (2009) Taxonomy of the fouling cheilostome bryozoans *Schizoporella unicornis* (Johnston) and *Schizoporella errata* (Waters). J Nat Hist 43 (35–36): 2227–2243.

Utinomi H (1967) Comments on some new and already know Cirripeds with emended taxa, with special reference to the parietal structure. Publ. Seto Marine Biol. Lab. XV(3): 199-237.

Vieira LM, Migotto AE, Winston JE (2008) Synopsis and annotated checklist of recent marine Bryozoa from Brazil. Zootaxa 1810: 1–39.

Young PS (1994) Superfamily Balanoidea Leach (Cirripedia, Balanomorpha) from the Brazilian Coast. Bol Mus Nac 356: 1-36.

Young PS (1998) Maxillopoda. Thecostraca. In: Young PS, editor. Catalogue of Crustacea of Brazil. Museu Nacional do Rio de Janeiro: Série de Livros 6. pp. 263-285.

Zullo VA (1992) *Balanus trigonus* Darwin (Cirripedia, Balaninae) in the Atlantic Basin: an introduced species? B Mar Sci 50 (1): 66-74.
